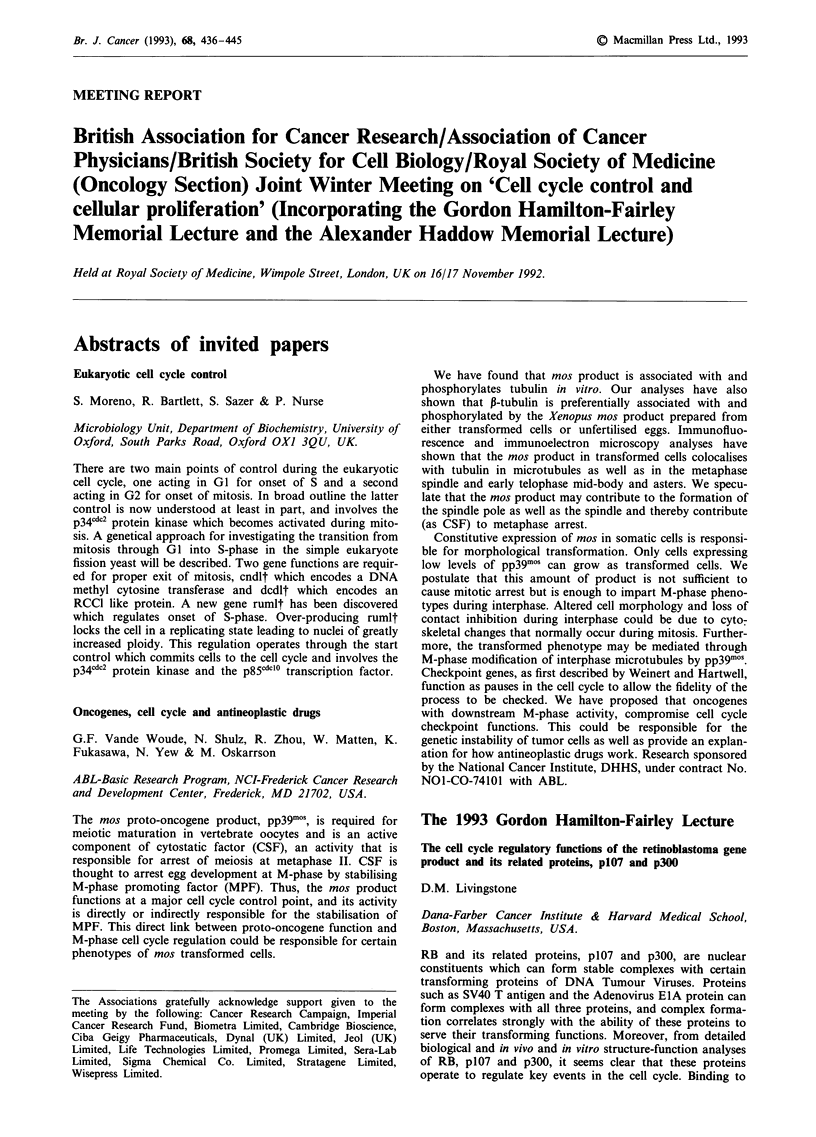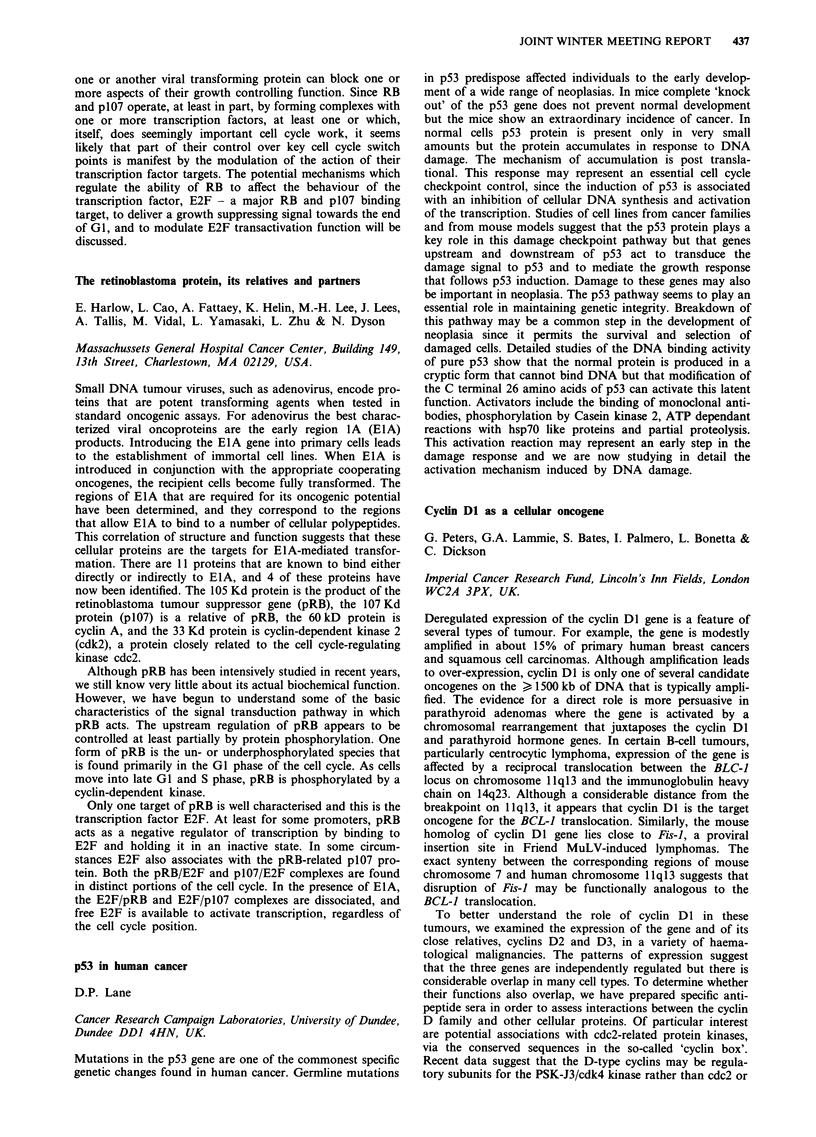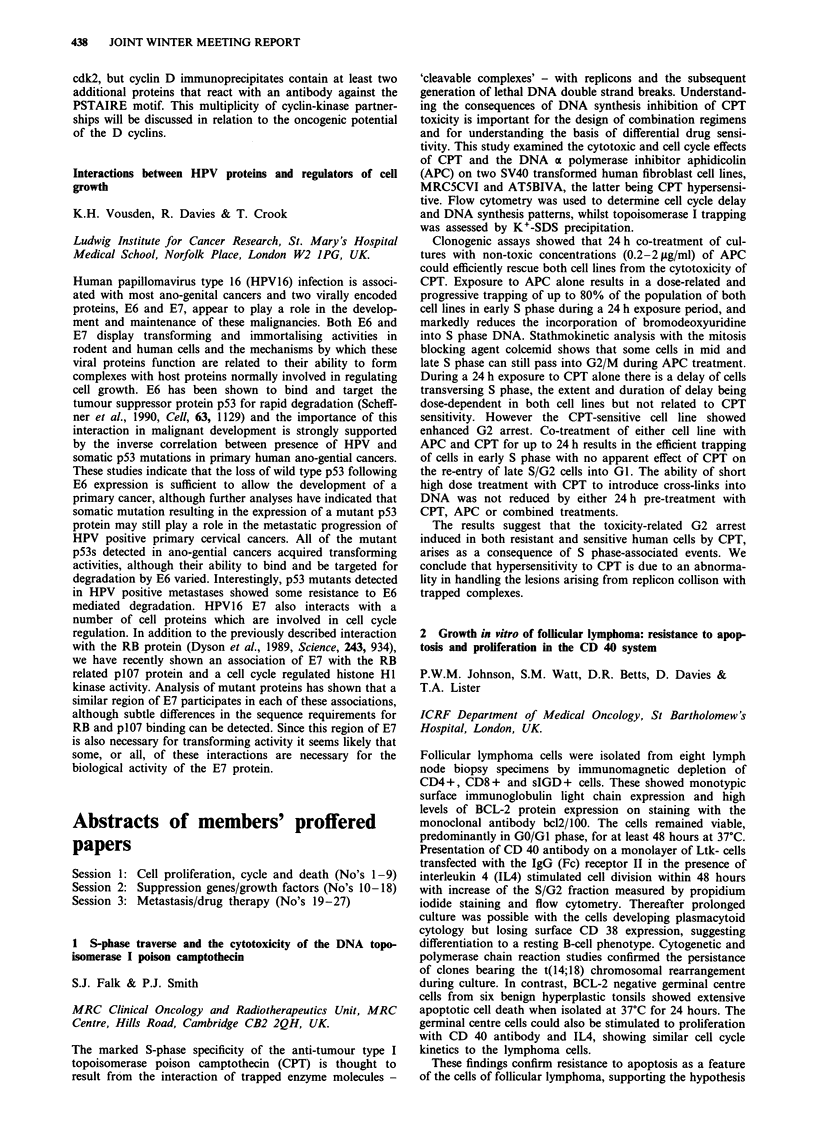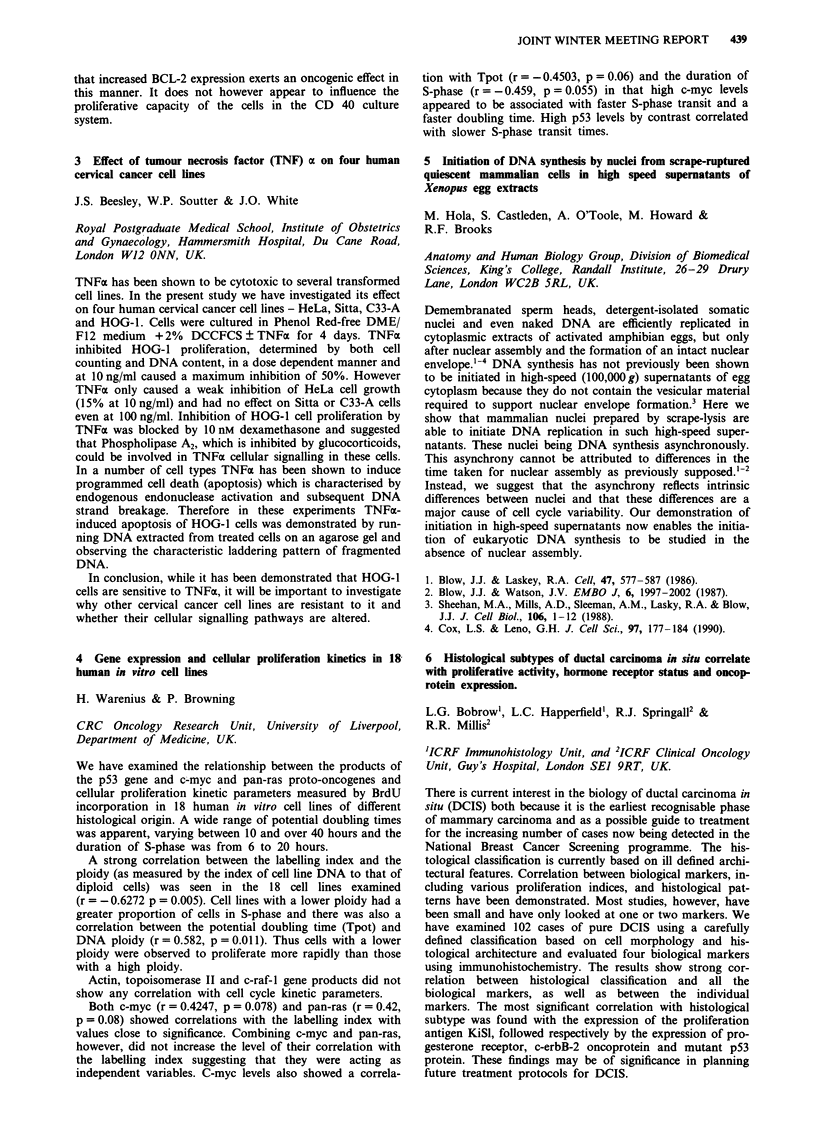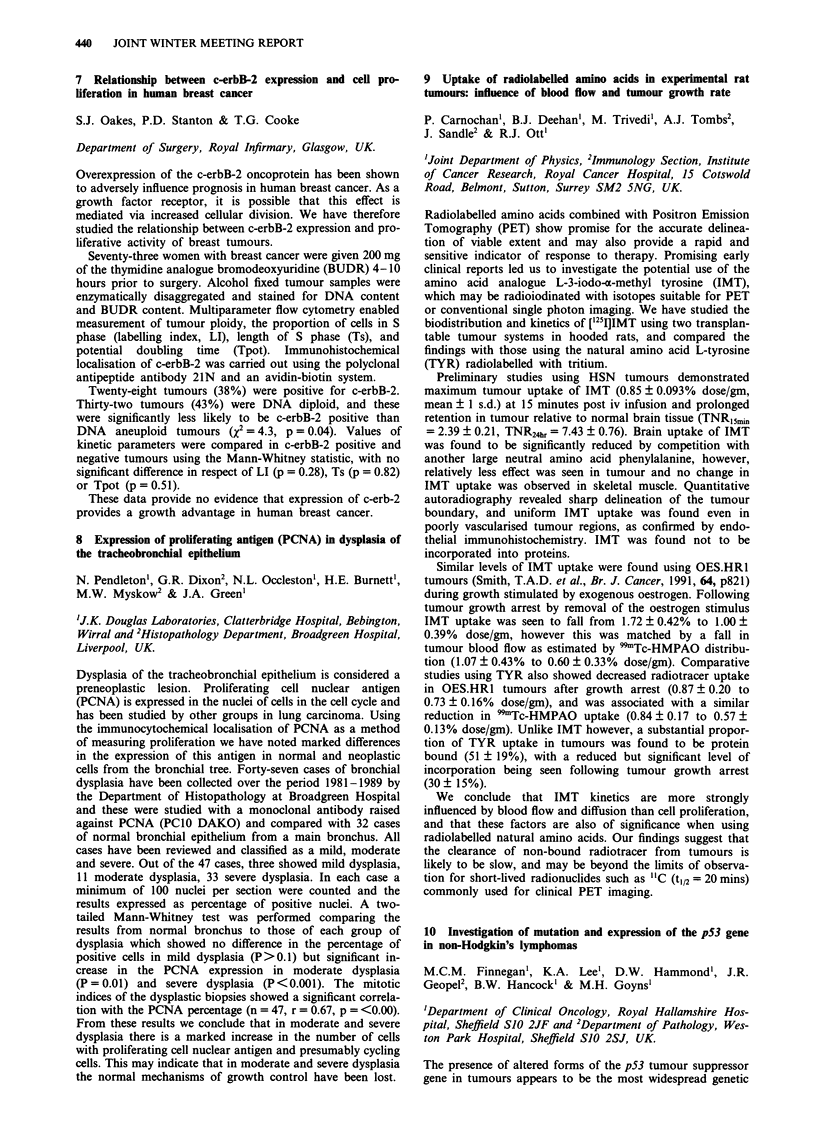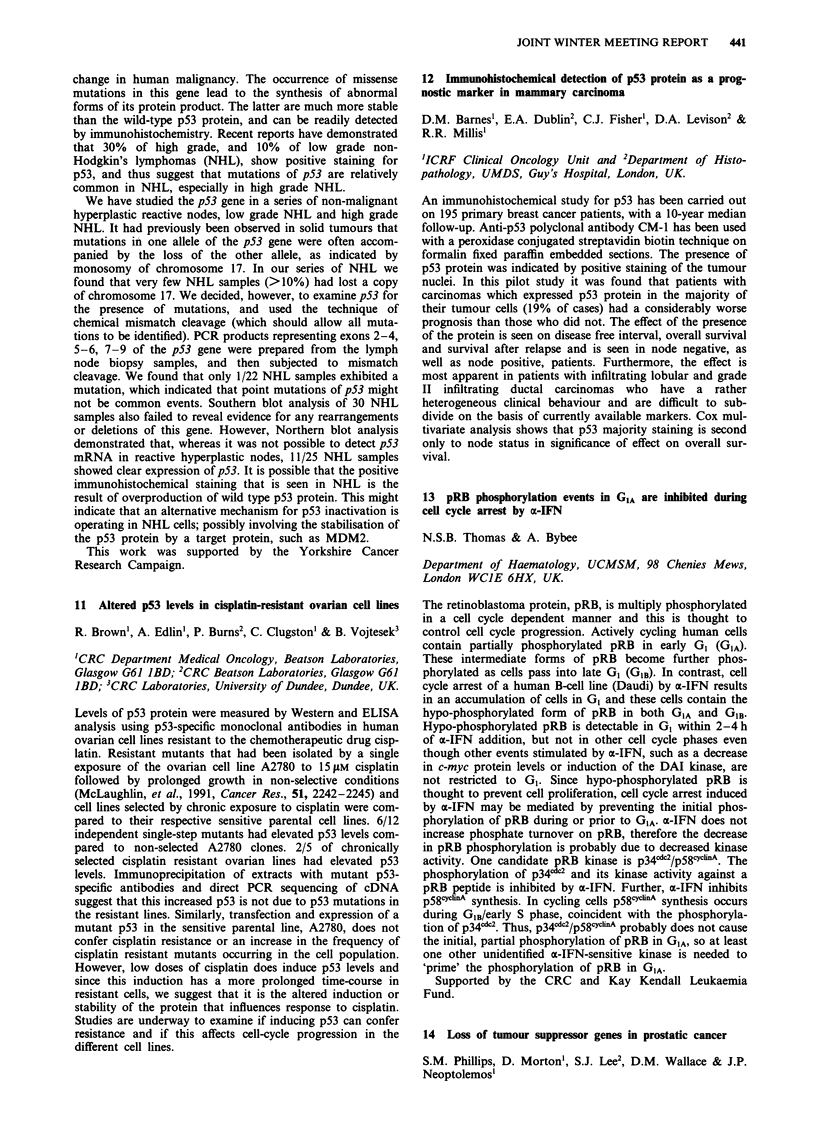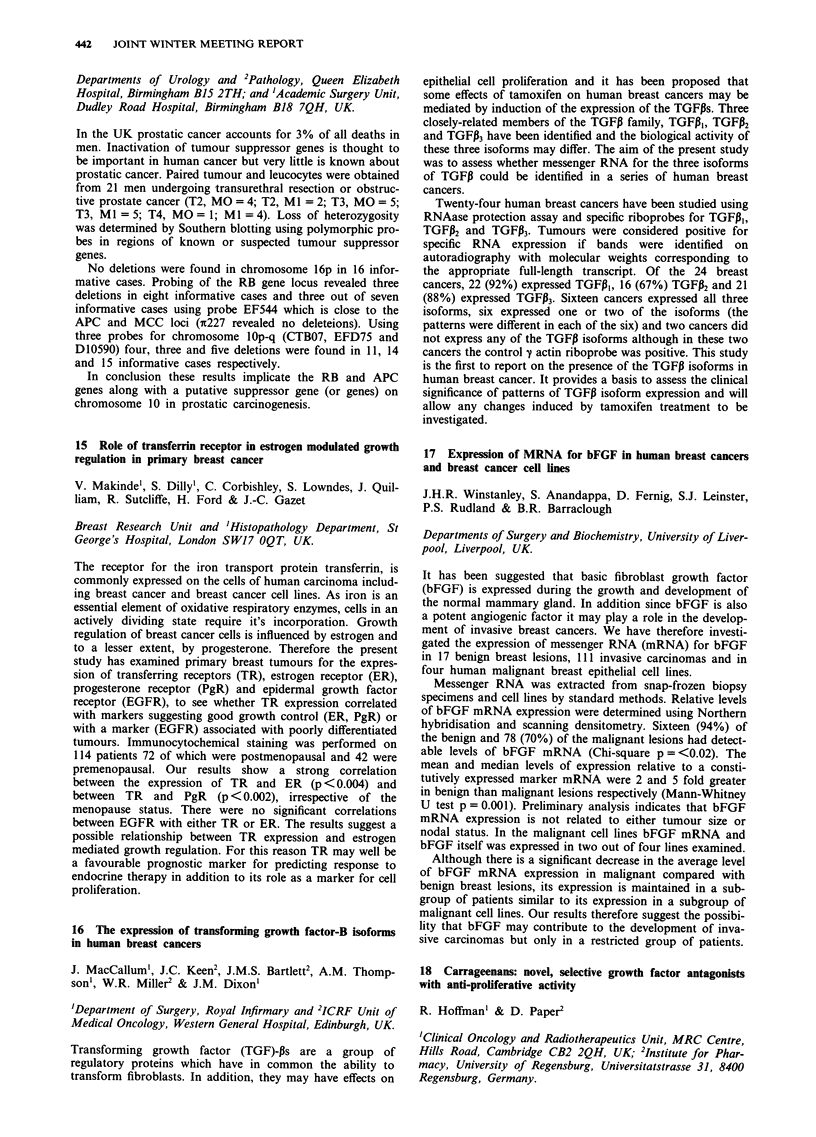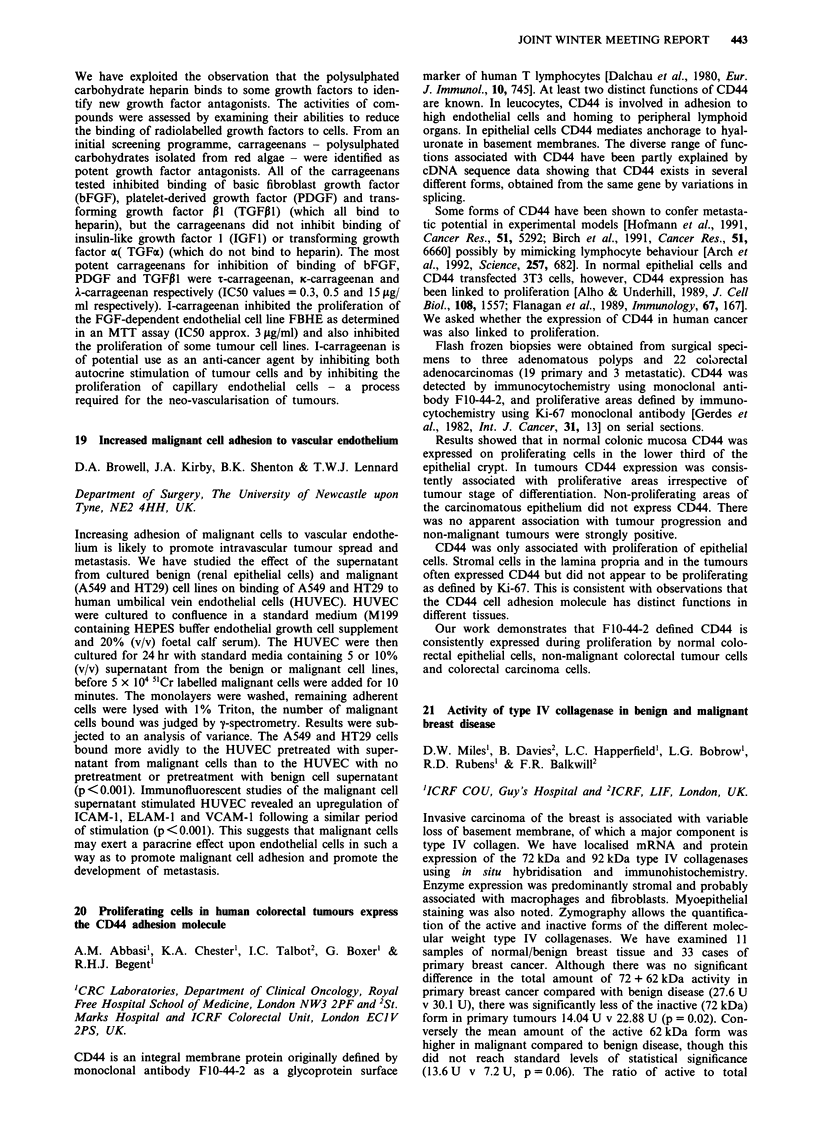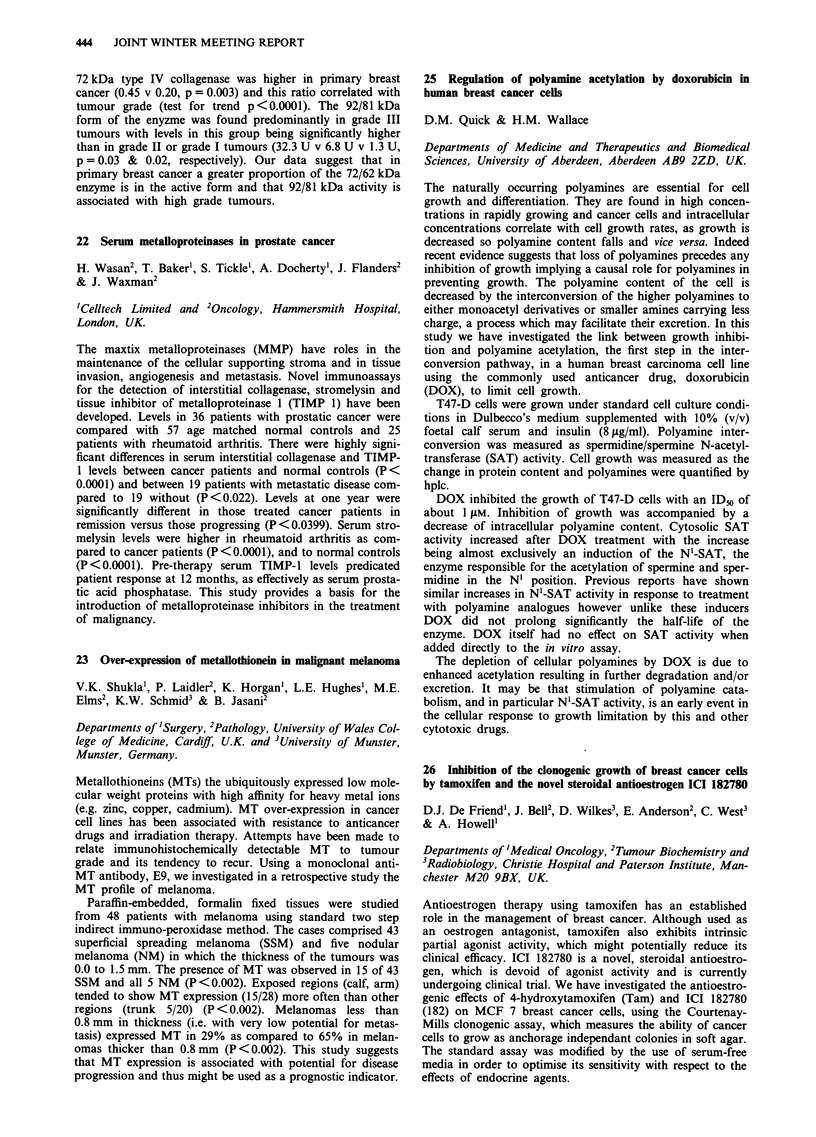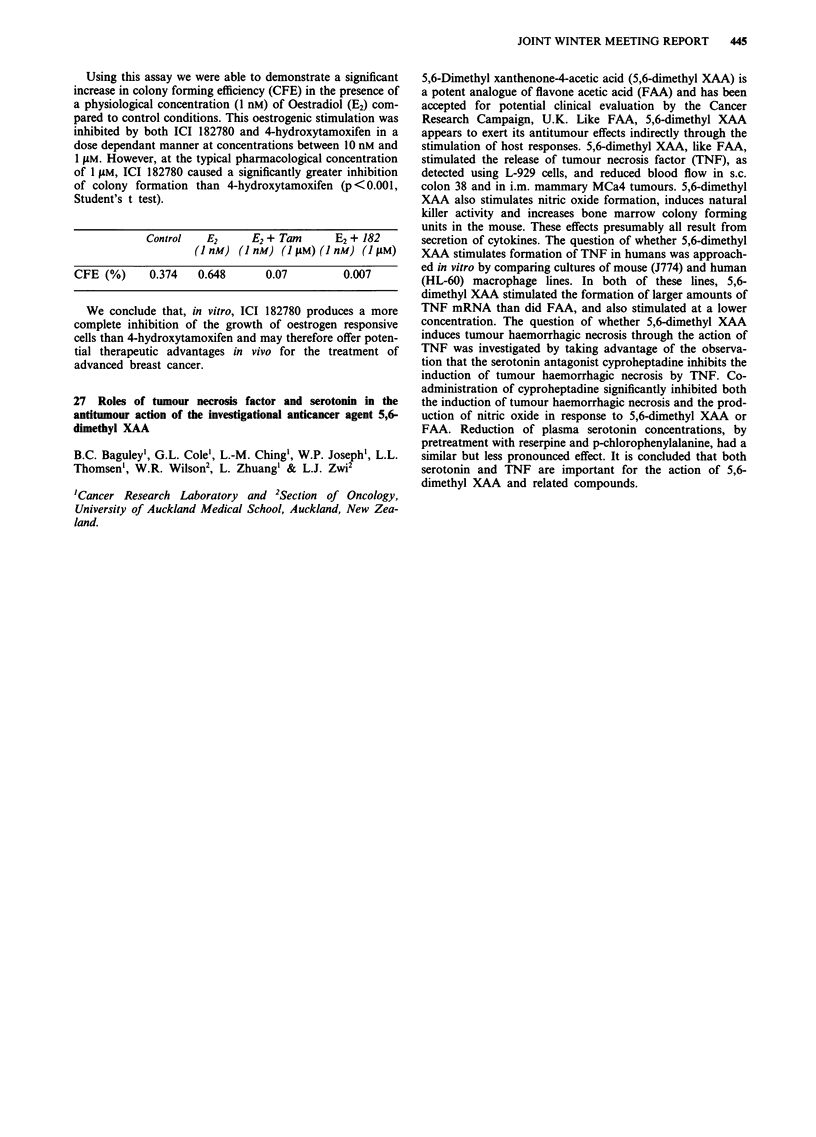# British Association for Cancer Research/ 'Association of Cancer Physicians/British Society for Cell Biology/Royal Society of Medicine (Oncology Section) Joint Winter Meeting on `Cell cycle control and cellular proliferation' (Incorporating the Gordon Hamilton-Fairley Memorial Lecture and the Alexander Haddow Memorial Lecture)

**Published:** 1993-08

**Authors:** 


 © Macmillan Press Ltd., 1993

MEETING REPORT

### British Association for Cancer Research/Association of Cancer Physicians/British Society for Cell Biology/Royal Society of Medicine (Oncology Section) Joint Winter Meeting on 'Cell cycle control and cellular proliferation' (Incorporating the Gordon Hamilton-Fairley Memorial Lecture and the Alexander Haddow Memorial Lecture)

*Held at Royal Society of Medicine, Wimpole Street, London, UK on 16/17 November 1992.*

### Abstracts of invited papers

### Eukaryotic cell cycle control

S. Moreno, R. Bartlett, S. Sazer & P. Nurse

*Microbiology Unit, Department of Biochemistry, University of Oxford, South Parks Road, Oxford OX1 3QU, UK.*

There are two main points of control during the eukaryotic cell cycle, one acting in G1 for onset of S and a second acting in G2 for onset of mitosis. In broad outline the latter control is now understood at least in part, and involves the $p34^{cdc2}$ protein kinase which becomes activated during mitosis. A genetical approach for investigating the transition from mitosis through G1 into S-phase in the simple eukaryote fission yeast will be described. Two gene functions are required for proper exit of mitosis, cndl† which encodes a DNA methyl cytosine transferase and dcdl† which encodes an RCCl like protein. A new gene ruml† has been discovered which regulates onset of S-phase. Over-producing ruml† locks the cell in a replicating state leading to nuclei of greatly increased ploidy. This regulation operates through the start control which commits cells to the cell cycle and involves the $p34^{cdc2}$ protein kinase and the $p85^{cdc10}$ transcription factor.

### Oncogenes, cell cycle and antineoplastic drugs

G.F. Vande Woude, N. Shulz, R. Zhou, W. Matten, K. Fukasawa, N. Yew & M. Oskarrson

*ABL-Basic Research Program, NCI-Frederick Cancer Research and Development Center, Frederick, MD 21702, USA.*

The *mos* proto-oncogene product, $pp39^{mos}$, is required for meiotic maturation in vertebrate oocytes and is an active component of cytostatic factor (CSF), an activity that is responsible for arrest of meiosis at metaphase II. CSF is thought to arrest egg development at M-phase by stabilising M-phase promoting factor (MPF). Thus, the *mos* product functions at a major cell cycle control point, and its activity is directly or indirectly responsible for the stabilisation of MPF. This direct link between proto-oncogene function and M-phase cell cycle regulation could be responsible for certain phenotypes of *mos* transformed cells.

We have found that *mos* product is associated with and phosphorylates tubulin *in vitro*. Our analyses have also shown that β-tubulin is preferentially associated with and phosphorylated by the *Xenopus mos* product prepared from either transformed cells or unfertilised eggs. Immunofluorescence and immunoelectron microscopy analyses have shown that the *mos* product in transformed cells colocalises with tubulin in microtubules as well as in the metaphase spindle and early telophase mid-body and asters. We speculate that the *mos* product may contribute to the formation of the spindle pole as well as the spindle and thereby contribute (as CSF) to metaphase arrest.

Constitutive expression of *mos* in somatic cells is responsible for morphological transformation. Only cells expressing low levels of $pp39^{mos}$ can grow as transformed cells. We postulate that this amount of product is not sufficient to cause mitotic arrest but is enough to impart M-phase phenotypes during interphase. Altered cell morphology and loss of contact inhibition during interphase could be due to cytoskeletal changes that normally occur during mitosis. Furthermore, the transformed phenotype may be mediated through M-phase modification of interphase microtubules by $pp39^{mos}$. Checkpoint genes, as first described by Weinert and Hartwell, function as pauses in the cell cycle to allow the fidelity of the process to be checked. We have proposed that oncogenes with downstream M-phase activity, compromise cell cycle checkpoint functions. This could be responsible for the genetic instability of tumor cells as well as provide an explanation for how antineoplastic drugs work. Research sponsored by the National Cancer Institute, DHHS, under contract No. NO1-CO-74101 with ABL.

### The 1993 Gordon Hamilton-Fairley Lecture

### The cell cycle regulatory functions of the retinoblastoma gene product and its related proteins, p107 and p300

D.M. Livingstone

*Dana-Farber Cancer Institute & Harvard Medical School, Boston, Massachusetts, USA.*

RB and its related proteins, p107 and p300, are nuclear constituents which can form stable complexes with certain transforming proteins of DNA Tumour Viruses. Proteins such as SV40 T antigen and the Adenovirus E1A protein can form complexes with all three proteins, and complex formation correlates strongly with the ability of these proteins to serve their transforming functions. Moreover, from detailed biological and *in vivo* and *in vitro* structure-function analyses of RB, p107 and p300, it seems clear that these proteins operate to regulate key events in the cell cycle. Binding to

The Associations gratefully acknowledge support given to the meeting by the following: Cancer Research Campaign, Imperial Cancer Research Fund, Biometra Limited, Cambridge Bioscience, Ciba Geigy Pharmaceuticals, Dynal (UK) Limited, Jeol (UK) Limited, Life Technologies Limited, Promega Limited, Sera-Lab Limited, Sigma Chemical Co. Limited, Stratagene Limited, Wisepress Limited.

one or another viral transforming protein can block one or more aspects of their growth controlling function. Since RB and p107 operate, at least in part, by forming complexes with one or more transcription factors, at least one or which, itself, does seemingly important cell cycle work, it seems likely that part of their control over key cell cycle switch points is manifest by the modulation of the action of their transcription factor targets. The potential mechanisms which regulate the ability of RB to affect the behaviour of the transcription factor, E2F – a major RB and p107 binding target, to deliver a growth suppressing signal towards the end of G1, and to modulate E2F transactivation function will be discussed.

**The retinoblastoma protein, its relatives and partners**

E. Harlow, L. Cao, A. Fattaey, K. Helin, M.-H. Lee, J. Lees, A. Tallis, M. Vidal, L. Yamasaki, L. Zhu & N. Dyson

*Massachussets General Hospital Cancer Center, Building 149, 13th Street, Charlestown, MA 02129, USA.*

Small DNA tumour viruses, such as adenovirus, encode proteins that are potent transforming agents when tested in standard oncogenic assays. For adenovirus the best characterized viral oncoproteins are the early region 1A (E1A) products. Introducing the E1A gene into primary cells leads to the establishment of immortal cell lines. When E1A is introduced in conjunction with the appropriate cooperating oncogenes, the recipient cells become fully transformed. The regions of E1A that are required for its oncogenic potential have been determined, and they correspond to the regions that allow E1A to bind to a number of cellular polypeptides. This correlation of structure and function suggests that these cellular proteins are the targets for E1A-mediated transformation. There are 11 proteins that are known to bind either directly or indirectly to E1A, and 4 of these proteins have now been identified. The 105 Kd protein is the product of the retinoblastoma tumour suppressor gene (pRB), the 107 Kd protein (p107) is a relative of pRB, the 60 kD protein is cyclin A, and the 33 Kd protein is cyclin-dependent kinase 2 (cdk2), a protein closely related to the cell cycle-regulating kinase cdc2.

Although pRB has been intensively studied in recent years, we still know very little about its actual biochemical function. However, we have begun to understand some of the basic characteristics of the signal transduction pathway in which pRB acts. The upstream regulation of pRB appears to be controlled at least partially by protein phosphorylation. One form of pRB is the un- or underphosphorylated species that is found primarily in the G1 phase of the cell cycle. As cells move into late G1 and S phase, pRB is phosphorylated by a cyclin-dependent kinase.

Only one target of pRB is well characterised and this is the transcription factor E2F. At least for some promoters, pRB acts as a negative regulator of transcription by binding to E2F and holding it in an inactive state. In some circumstances E2F also associates with the pRB-related p107 protein. Both the pRB/E2F and p107/E2F complexes are found in distinct portions of the cell cycle. In the presence of E1A, the E2F/pRB and E2F/p107 complexes are dissociated, and free E2F is available to activate transcription, regardless of the cell cycle position.

**p53 in human cancer**

D.P. Lane

*Cancer Research Campaign Laboratories, University of Dundee, Dundee DD1 4HN, UK.*

Mutations in the p53 gene are one of the commonest specific genetic changes found in human cancer. Germline mutations in p53 predispose affected individuals to the early development of a wide range of neoplasias. In mice complete 'knock out' of the p53 gene does not prevent normal development but the mice show an extraordinary incidence of cancer. In normal cells p53 protein is present only in very small amounts but the protein accumulates in response to DNA damage. The mechanism of accumulation is post translational. This response may represent an essential cell cycle checkpoint control, since the induction of p53 is associated with an inhibition of cellular DNA synthesis and activation of the transcription. Studies of cell lines from cancer families and from mouse models suggest that the p53 protein plays a key role in this damage checkpoint pathway but that genes upstream and downstream of p53 act to transduce the damage signal to p53 and to mediate the growth response that follows p53 induction. Damage to these genes may also be important in neoplasia. The p53 pathway seems to play an essential role in maintaining genetic integrity. Breakdown of this pathway may be a common step in the development of neoplasia since it permits the survival and selection of damaged cells. Detailed studies of the DNA binding activity of pure p53 show that the normal protein is produced in a cryptic form that cannot bind DNA but that modification of the C terminal 26 amino acids of p53 can activate this latent function. Activators include the binding of monoclonal antibodies, phosphorylation by Casein kinase 2, ATP dependant reactions with hsp70 like proteins and partial proteolysis. This activation reaction may represent an early step in the damage response and we are now studying in detail the activation mechanism induced by DNA damage.

**Cyclin D1 as a cellular oncogene**

G. Peters, G.A. Lammie, S. Bates, I. Palmero, L. Bonetta & C. Dickson

*Imperial Cancer Research Fund, Lincoln's Inn Fields, London WC2A 3PX, UK.*

Deregulated expression of the cyclin D1 gene is a feature of several types of tumour. For example, the gene is modestly amplified in about 15% of primary human breast cancers and squamous cell carcinomas. Although amplification leads to over-expression, cyclin D1 is only one of several candidate oncogenes on the $\geqslant$1500 kb of DNA that is typically amplified. The evidence for a direct role is more persuasive in parathyroid adenomas where the gene is activated by a chromosomal rearrangement that juxtaposes the cyclin D1 and parathyroid hormone genes. In certain B-cell tumours, particularly centrocytic lymphoma, expression of the gene is affected by a reciprocal translocation between the *BLC-1* locus on chromosome 11q13 and the immunoglobulin heavy chain on 14q23. Although a considerable distance from the breakpoint on 11q13, it appears that cyclin D1 is the target oncogene for the *BCL-1* translocation. Similarly, the mouse homolog of cyclin D1 gene lies close to *Fis-1*, a proviral insertion site in Friend MuLV-induced lymphomas. The exact synteny between the corresponding regions of mouse chromosome 7 and human chromosome 11q13 suggests that disruption of *Fis-1* may be functionally analogous to the *BCL-1* translocation.

To better understand the role of cyclin D1 in these tumours, we examined the expression of the gene and of its close relatives, cyclins D2 and D3, in a variety of haematological malignancies. The patterns of expression suggest that the three genes are independently regulated but there is considerable overlap in many cell types. To determine whether their functions also overlap, we have prepared specific anti-peptide sera in order to assess interactions between the cyclin D family and other cellular proteins. Of particular interest are potential associations with cdc2-related protein kinases, via the conserved sequences in the so-called 'cyclin box'. Recent data suggest that the D-type cyclins may be regulatory subunits for the PSK-J3/cdk4 kinase rather than cdc2 or cdk2, but cyclin D immunoprecipitates contain at least two additional proteins that react with an antibody against the PSTAIRE motif. This multiplicity of cyclin-kinase partnerships will be discussed in relation to the oncogenic potential of the D cyclins.

**Interactions between HPV proteins and regulators of cell growth**

K.H. Vousden, R. Davies & T. Crook

*Ludwig Institute for Cancer Research, St. Mary's Hospital Medical School, Norfolk Place, London W2 1PG, UK.*

Human papillomavirus type 16 (HPV16) infection is associated with most ano-genital cancers and two virally encoded proteins, E6 and E7, appear to play a role in the development and maintenance of these malignancies. Both E6 and E7 display transforming and immortalising activities in rodent and human cells and the mechanisms by which these viral proteins function are related to their ability to form complexes with host proteins normally involved in regulating cell growth. E6 has been shown to bind and target the tumour suppressor protein p53 for rapid degradation (Scheffner *et al.*, 1990, *Cell*, **63**, 1129) and the importance of this interaction in malignant development is strongly supported by the inverse correlation between presence of HPV and somatic p53 mutations in primary human ano-gential cancers. These studies indicate that the loss of wild type p53 following E6 expression is sufficient to allow the development of a primary cancer, although further analyses have indicated that somatic mutation resulting in the expression of a mutant p53 protein may still play a role in the metastatic progression of HPV positive primary cervical cancers. All of the mutant p53s detected in ano-gential cancers acquired transforming activities, although their ability to bind and be targeted for degradation by E6 varied. Interestingly, p53 mutants detected in HPV positive metastases showed some resistance to E6 mediated degradation. HPV16 E7 also interacts with a number of cell proteins which are involved in cell cycle regulation. In addition to the previously described interaction with the RB protein (Dyson *et al.*, 1989, *Science*, **243**, 934), we have recently shown an association of E7 with the RB related p107 protein and a cell cycle regulated histone H1 kinase activity. Analysis of mutant proteins has shown that a similar region of E7 participates in each of these associations, although subtle differences in the sequence requirements for RB and p107 binding can be detected. Since this region of E7 is also necessary for transforming activity it seems likely that some, or all, of these interactions are necessary for the biological activity of the E7 protein.

### Abstracts of members' proffered papers

Session 1: Cell proliferation, cycle and death (No's 1–9)
Session 2: Suppression genes/growth factors (No's 10–18)
Session 3: Metastasis/drug therapy (No's 19–27)

**1 S-phase traverse and the cytotoxicity of the DNA topoisomerase I poison camptothecin**

S.J. Falk & P.J. Smith

*MRC Clinical Oncology and Radiotherapeutics Unit, MRC Centre, Hills Road, Cambridge CB2 2QH, UK.*

The marked S-phase specificity of the anti-tumour type I topoisomerase poison camptothecin (CPT) is thought to result from the interaction of trapped enzyme molecules – 'cleavable complexes' – with replicons and the subsequent generation of lethal DNA double strand breaks. Understanding the consequences of DNA synthesis inhibition of CPT toxicity is important for the design of combination regimens and for understanding the basis of differential drug sensitivity. This study examined the cytotoxic and cell cycle effects of CPT and the DNA α polymerase inhibitor aphidicolin (APC) on two SV40 transformed human fibroblast cell lines, MRC5CVI and AT5BIVA, the latter being CPT hypersensitive. Flow cytometry was used to determine cell cycle delay and DNA synthesis patterns, whilst topoisomerase I trapping was assessed by $K^+$-SDS precipitation.

Clonogenic assays showed that 24 h co-treatment of cultures with non-toxic concentrations (0.2–2 μg/ml) of APC could efficiently rescue both cell lines from the cytotoxicity of CPT. Exposure to APC alone results in a dose-related and progressive trapping of up to 80% of the population of both cell lines in early S phase during a 24 h exposure period, and markedly reduces the incorporation of bromodeoxyuridine into S phase DNA. Stathmokinetic analysis with the mitosis blocking agent colcemid shows that some cells in mid and late S phase can still pass into G2/M during APC treatment. During a 24 h exposure to CPT alone there is a delay of cells transversing S phase, the extent and duration of delay being dose-dependent in both cell lines but not related to CPT sensitivity. However the CPT-sensitive cell line showed enhanced G2 arrest. Co-treatment of either cell line with APC and CPT for up to 24 h results in the efficient trapping of cells in early S phase with no apparent effect of CPT on the re-entry of late S/G2 cells into G1. The ability of short high dose treatment with CPT to introduce cross-links into DNA was not reduced by either 24 h pre-treatment with CPT, APC or combined treatments.

The results suggest that the toxicity-related G2 arrest induced in both resistant and sensitive human cells by CPT, arises as a consequence of S phase-associated events. We conclude that hypersensitivity to CPT is due to an abnormality in handling the lesions arising from replicon collison with trapped complexes.

**2 Growth *in vitro* of follicular lymphoma: resistance to apoptosis and proliferation in the CD 40 system**

P.W.M. Johnson, S.M. Watt, D.R. Betts, D. Davies & T.A. Lister

*ICRF Department of Medical Oncology, St Bartholomew's Hospital, London, UK.*

Follicular lymphoma cells were isolated from eight lymph node biopsy specimens by immunomagnetic depletion of CD4+, CD8+ and sIGD+ cells. These showed monotypic surface immunoglobulin light chain expression and high levels of BCL-2 protein expression on staining with the monoclonal antibody bcl2/100. The cells remained viable, predominantly in G0/G1 phase, for at least 48 hours at 37°C. Presentation of CD 40 antibody on a monolayer of Ltk- cells transfected with the IgG (Fc) receptor II in the presence of interleukin 4 (IL4) stimulated cell division within 48 hours with increase of the S/G2 fraction measured by propidium iodide staining and flow cytometry. Thereafter prolonged culture was possible with the cells developing plasmacytoid cytology but losing surface CD 38 expression, suggesting differentiation to a resting B-cell phenotype. Cytogenetic and polymerase chain reaction studies confirmed the persistance of clones bearing the t(14;18) chromosomal rearrangement during culture. In contrast, BCL-2 negative germinal centre cells from six benign hyperplastic tonsils showed extensive apoptotic cell death when isolated at 37°C for 24 hours. The germinal centre cells could also be stimulated to proliferation with CD 40 antibody and IL4, showing similar cell cycle kinetics to the lymphoma cells.

These findings confirm resistance to apoptosis as a feature of the cells of follicular lymphoma, supporting the hypothesis that increased BCL-2 expression exerts an oncogenic effect in this manner. It does not however appear to influence the proliferative capacity of the cells in the CD 40 culture system.

**3 Effect of tumour necrosis factor (TNF) α on four human cervical cancer cell lines**

J.S. Beesley, W.P. Soutter & J.O. White

*Royal Postgraduate Medical School, Institute of Obstetrics and Gynaecology, Hammersmith Hospital, Du Cane Road, London W12 0NN, UK.*

TNFα has been shown to be cytotoxic to several transformed cell lines. In the present study we have investigated its effect on four human cervical cancer cell lines - HeLa, Sitta, C33-A and HOG-1. Cells were cultured in Phenol Red-free DME/F12 medium +2% DCCFCS ± TNFα for 4 days. TNFα inhibited HOG-1 proliferation, determined by both cell counting and DNA content, in a dose dependent manner and at 10 ng/ml caused a maximum inhibition of 50%. However TNFα only caused a weak inhibition of HeLa cell growth (15% at 10 ng/ml) and had no effect on Sitta or C33-A cells even at 100 ng/ml. Inhibition of HOG-1 cell proliferation by TNFα was blocked by 10 nM dexamethasone and suggested that Phospholipase $A_2$, which is inhibited by glucocorticoids, could be involved in TNFα cellular signalling in these cells. In a number of cell types TNFα has been shown to induce programmed cell death (apoptosis) which is characterised by endogenous endonuclease activation and subsequent DNA strand breakage. Therefore in these experiments TNFα-induced apoptosis of HOG-1 cells was demonstrated by running DNA extracted from treated cells on an agarose gel and observing the characteristic laddering pattern of fragmented DNA.

In conclusion, while it has been demonstrated that HOG-1 cells are sensitive to TNFα, it will be important to investigate why other cervical cancer cell lines are resistant to it and whether their cellular signalling pathways are altered.

**4 Gene expression and cellular proliferation kinetics in 18 human *in vitro* cell lines**

H. Warenius & P. Browning

*CRC Oncology Research Unit, University of Liverpool, Department of Medicine, UK.*

We have examined the relationship between the products of the p53 gene and c-myc and pan-ras proto-oncogenes and cellular proliferation kinetic parameters measured by BrdU incorporation in 18 human *in vitro* cell lines of different histological origin. A wide range of potential doubling times was apparent, varying between 10 and over 40 hours and the duration of S-phase was from 6 to 20 hours.

A strong correlation between the labelling index and the ploidy (as measured by the index of cell line DNA to that of diploid cells) was seen in the 18 cell lines examined ($r = -0.6272$ $p = 0.005$). Cell lines with a lower ploidy had a greater proportion of cells in S-phase and there was also a correlation between the potential doubling time (Tpot) and DNA ploidy ($r = 0.582$, $p = 0.011$). Thus cells with a lower ploidy were observed to proliferate more rapidly than those with a high ploidy.

Actin, topoisomerase II and c-raf-1 gene products did not show any correlation with cell cycle kinetic parameters.

Both c-myc ($r = 0.4247$, $p = 0.078$) and pan-ras ($r = 0.42$, $p = 0.08$) showed correlations with the labelling index with values close to significance. Combining c-myc and pan-ras, however, did not increase the level of their correlation with the labelling index suggesting that they were acting as independent variables. C-myc levels also showed a correlation with Tpot ($r = -0.4503$, $p = 0.06$) and the duration of S-phase ($r = -0.459$, $p = 0.055$) in that high c-myc levels appeared to be associated with faster S-phase transit and a faster doubling time. High p53 levels by contrast correlated with slower S-phase transit times.

**5 Initiation of DNA synthesis by nuclei from scrape-ruptured quiescent mammalian cells in high speed supernatants of *Xenopus* egg extracts**

M. Hola, S. Castleden, A. O'Toole, M. Howard & R.F. Brooks

*Anatomy and Human Biology Group, Division of Biomedical Sciences, King's College, Randall Institute, 26–29 Drury Lane, London WC2B 5RL, UK.*

Demembranated sperm heads, detergent-isolated somatic nuclei and even naked DNA are efficiently replicated in cytoplasmic extracts of activated amphibian eggs, but only after nuclear assembly and the formation of an intact nuclear envelope.[1–4] DNA synthesis has not previously been shown to be initiated in high-speed (100,000 *g*) supernatants of egg cytoplasm because they do not contain the vesicular material required to support nuclear envelope formation.[3] Here we show that mammalian nuclei prepared by scrape-lysis are able to initiate DNA replication in such high-speed supernatants. These nuclei being DNA synthesis asynchronously. This asynchrony cannot be attributed to differences in the time taken for nuclear assembly as previously supposed.[1–2] Instead, we suggest that the asynchrony reflects intrinsic differences between nuclei and that these differences are a major cause of cell cycle variability. Our demonstration of initiation in high-speed supernatants now enables the initiation of eukaryotic DNA synthesis to be studied in the absence of nuclear assembly.

1. Blow, J.J. & Laskey, R.A. *Cell*, **47**, 577–587 (1986).
2. Blow, J.J. & Watson, J.V. *EMBO J*, **6**, 1997–2002 (1987).
3. Sheehan, M.A., Mills, A.D., Sleeman, A.M., Lasky, R.A. & Blow, J.J. *J. Cell Biol.*, **106**, 1–12 (1988).
4. Cox, L.S. & Leno, G.H. *J. Cell Sci.*, **97**, 177–184 (1990).

**6 Histological subtypes of ductal carcinoma *in situ* correlate with proliferative activity, hormone receptor status and oncoprotein expression.**

L.G. Bobrow[1], L.C. Happerfield[1], R.J. Springall[2] & R.R. Millis[2]

[1]*ICRF Immunohistology Unit, and* [2]*ICRF Clinical Oncology Unit, Guy's Hospital, London SE1 9RT, UK.*

There is current interest in the biology of ductal carcinoma *in situ* (DCIS) both because it is the earliest recognisable phase of mammary carcinoma and as a possible guide to treatment for the increasing number of cases now being detected in the National Breast Cancer Screening programme. The histological classification is currently based on ill defined architectural features. Correlation between biological markers, including various proliferation indices, and histological patterns have been demonstrated. Most studies, however, have been small and have only looked at one or two markers. We have examined 102 cases of pure DCIS using a carefully defined classification based on cell morphology and histological architecture and evaluated four biological markers using immunohistochemistry. The results show strong correlation between histological classification and all the biological markers, as well as between the individual markers. The most significant correlation with histological subtype was found with the expression of the proliferation antigen KiSl, followed respectively by the expression of progesterone receptor, c-erbB-2 oncoprotein and mutant p53 protein. These findings may be of significance in planning future treatment protocols for DCIS.

**7 Relationship between c-erbB-2 expression and cell proliferation in human breast cancer**

S.J. Oakes, P.D. Stanton & T.G. Cooke

*Department of Surgery, Royal Infirmary, Glasgow, UK.*

Overexpression of the c-erbB-2 oncoprotein has been shown to adversely influence prognosis in human breast cancer. As a growth factor receptor, it is possible that this effect is mediated via increased cellular division. We have therefore studied the relationship between c-erbB-2 expression and proliferative activity of breast tumours.

Seventy-three women with breast cancer were given 200 mg of the thymidine analogue bromodeoxyuridine (BUDR) 4–10 hours prior to surgery. Alcohol fixed tumour samples were enzymatically disaggregated and stained for DNA content and BUDR content. Multiparameter flow cytometry enabled measurement of tumour ploidy, the proportion of cells in S phase (labelling index, LI), length of S phase (Ts), and potential doubling time (Tpot). Immunohistochemical localisation of c-erbB-2 was carried out using the polyclonal antipeptide antibody 21N and an avidin-biotin system.

Twenty-eight tumours (38%) were positive for c-erbB-2. Thirty-two tumours (43%) were DNA diploid, and these were significantly less likely to be c-erbB-2 positive than DNA aneuploid tumours ($\chi^2 = 4.3$, $p = 0.04$). Values of kinetic parameters were compared in c-erbB-2 positive and negative tumours using the Mann-Whitney statistic, with no significant difference in respect of LI ($p = 0.28$), Ts ($p = 0.82$) or Tpot ($p = 0.51$).

These data provide no evidence that expression of c-erb-2 provides a growth advantage in human breast cancer.

**8 Expression of proliferating antigen (PCNA) in dysplasia of the tracheobronchial epithelium**

N. Pendleton[1], G.R. Dixon[2], N.L. Occleston[1], H.E. Burnett[1], M.W. Myskow[2] & J.A. Green[1]

*[1]J.K. Douglas Laboratories, Clatterbridge Hospital, Bebington, Wirral and [2]Histopathology Department, Broadgreen Hospital, Liverpool, UK.*

Dysplasia of the tracheobronchial epithelium is considered a preneoplastic lesion. Proliferating cell nuclear antigen (PCNA) is expressed in the nuclei of cells in the cell cycle and has been studied by other groups in lung carcinoma. Using the immunocytochemical localisation of PCNA as a method of measuring proliferation we have noted marked differences in the expression of this antigen in normal and neoplastic cells from the bronchial tree. Forty-seven cases of bronchial dysplasia have been collected over the period 1981–1989 by the Department of Histopathology at Broadgreen Hospital and these were studied with a monoclonal antibody raised against PCNA (PC10 DAKO) and compared with 32 cases of normal bronchial epithelium from a main bronchus. All cases have been reviewed and classified as a mild, moderate and severe. Out of the 47 cases, three showed mild dysplasia, 11 moderate dysplasia, 33 severe dysplasia. In each case a minimum of 100 nuclei per section were counted and the results expressed as percentage of positive nuclei. A two-tailed Mann-Whitney test was performed comparing the results from normal bronchus to those of each group of dysplasia which showed no difference in the percentage of positive cells in mild dysplasia ($P > 0.1$) but significant increase in the PCNA expression in moderate dysplasia ($P = 0.01$) and severe dysplasia ($P < 0.001$). The mitotic indices of the dysplastic biopsies showed a significant correlation with the PCNA percentage ($n = 47$, $r = 0.67$, $p = < 0.00$). From these results we conclude that in moderate and severe dysplasia there is a marked increase in the number of cells with proliferating cell nuclear antigen and presumably cycling cells. This may indicate that in moderate and severe dysplasia the normal mechanisms of growth control have been lost.

**9 Uptake of radiolabelled amino acids in experimental rat tumours: influence of blood flow and tumour growth rate**

P. Carnochan[1], B.J. Deehan[1], M. Trivedi[1], A.J. Tombs[2], J. Sandle[2] & R.J. Ott[1]

*[1]Joint Department of Physics, [2]Immunology Section, Institute of Cancer Research, Royal Cancer Hospital, 15 Cotswold Road, Belmont, Sutton, Surrey SM2 5NG, UK.*

Radiolabelled amino acids combined with Positron Emission Tomography (PET) show promise for the accurate delineation of viable extent and may also provide a rapid and sensitive indicator of response to therapy. Promising early clinical reports led us to investigate the potential use of the amino acid analogue L-3-iodo-α-methyl tyrosine (IMT), which may be radioiodinated with isotopes suitable for PET or conventional single photon imaging. We have studied the biodistribution and kinetics of [$^{125}$I]IMT using two transplantable tumour systems in hooded rats, and compared the findings with those using the natural amino acid L-tyrosine (TYR) radiolabelled with tritium.

Preliminary studies using HSN tumours demonstrated maximum tumour uptake of IMT (0.85 ± 0.093% dose/gm, mean ± 1 s.d.) at 15 minutes post iv infusion and prolonged retention in tumour relative to normal brain tissue ($TNR_{15min} = 2.39 \pm 0.21$, $TNR_{24hr} = 7.43 \pm 0.76$). Brain uptake of IMT was found to be significantly reduced by competition with another large neutral amino acid phenylalanine, however, relatively less effect was seen in tumour and no change in IMT uptake was observed in skeletal muscle. Quantitative autoradiography revealed sharp delineation of the tumour boundary, and uniform IMT uptake was found even in poorly vascularised tumour regions, as confirmed by endothelial immunohistochemistry. IMT was found not to be incorporated into proteins.

Similar levels of IMT uptake were found using OES.HR1 tumours (Smith, T.A.D. *et al.*, *Br. J. Cancer*, 1991, **64**, p821) during growth stimulated by exogenous oestrogen. Following tumour growth arrest by removal of the oestrogen stimulus IMT uptake was seen to fall from 1.72 ± 0.42% to 1.00 ± 0.39% dose/gm, however this was matched by a fall in tumour blood flow as estimated by $^{99m}$Tc-HMPAO distribution (1.07 ± 0.43% to 0.60 ± 0.33% dose/gm). Comparative studies using TYR also showed decreased radiotracer uptake in OES.HR1 tumours after growth arrest (0.87 ± 0.20 to 0.73 ± 0.16% dose/gm), and was associated with a similar reduction in $^{99m}$Tc-HMPAO uptake (0.84 ± 0.17 to 0.57 ± 0.13% dose/gm). Unlike IMT however, a substantial proportion of TYR uptake in tumours was found to be protein bound (51 ± 19%), with a reduced but significant level of incorporation being seen following tumour growth arrest (30 ± 15%).

We conclude that IMT kinetics are more strongly influenced by blood flow and diffusion than cell proliferation, and that these factors are also of significance when using radiolabelled natural amino acids. Our findings suggest that the clearance of non-bound radiotracer from tumours is likely to be slow, and may be beyond the limits of observation for short-lived radionuclides such as $^{11}$C ($t_{1/2} = 20$ mins) commonly used for clinical PET imaging.

**10 Investigation of mutation and expression of the *p53* gene in non-Hodgkin's lymphomas**

M.C.M. Finnegan[1], K.A. Lee[1], D.W. Hammond[1], J.R. Geopel[2], B.W. Hancock[1] & M.H. Goyns[1]

*[1]Department of Clinical Oncology, Royal Hallamshire Hospital, Sheffield S10 2JF and [2]Department of Pathology, Weston Park Hospital, Sheffield S10 2SJ, UK.*

The presence of altered forms of the *p53* tumour suppressor gene in tumours appears to be the most widespread genetic change in human malignancy. The occurrence of missense mutations in this gene lead to the synthesis of abnormal forms of its protein product. The latter are much more stable than the wild-type p53 protein, and can be readily detected by immunohistochemistry. Recent reports have demonstrated that 30% of high grade, and 10% of low grade non-Hodgkin's lymphomas (NHL), show positive staining for p53, and thus suggest that mutations of *p53* are relatively common in NHL, especially in high grade NHL.

We have studied the *p53* gene in a series of non-malignant hyperplastic reactive nodes, low grade NHL and high grade NHL. It had previously been observed in solid tumours that mutations in one allele of the *p53* gene were often accompanied by the loss of the other allele, as indicated by monosomy of chromosome 17. In our series of NHL we found that very few NHL samples (>10%) had lost a copy of chromosome 17. We decided, however, to examine *p53* for the presence of mutations, and used the technique of chemical mismatch cleavage (which should allow all mutations to be identified). PCR products representing exons 2–4, 5–6, 7–9 of the *p53* gene were prepared from the lymph node biopsy samples, and then subjected to mismatch cleavage. We found that only 1/22 NHL samples exhibited a mutation, which indicated that point mutations of *p53* might not be common events. Southern blot analysis of 30 NHL samples also failed to reveal evidence for any rearrangements or deletions of this gene. However, Northern blot analysis demonstrated that, whereas it was not possible to detect *p53* mRNA in reactive hyperplastic nodes, 11/25 NHL samples showed clear expression of *p53*. It is possible that the positive immunohistochemical staining that is seen in NHL is the result of overproduction of wild type p53 protein. This might indicate that an alternative mechanism for p53 inactivation is operating in NHL cells; possibly involving the stabilisation of the p53 protein by a target protein, such as MDM2.

This work was supported by the Yorkshire Cancer Research Campaign.

### 11 Altered p53 levels in cisplatin-resistant ovarian cell lines

R. Brown[1], A. Edlin[1], P. Burns[2], C. Clugston[1] & B. Vojtesek[3]

*[1]CRC Department Medical Oncology, Beatson Laboratories, Glasgow G61 1BD; [2]CRC Beatson Laboratories, Glasgow G61 1BD; [3]CRC Laboratories, University of Dundee, Dundee, UK.*

Levels of p53 protein were measured by Western and ELISA analysis using p53-specific monoclonal antibodies in human ovarian cell lines resistant to the chemotherapeutic drug cisplatin. Resistant mutants that had been isolated by a single exposure of the ovarian cell line A2780 to 15 μM cisplatin followed by prolonged growth in non-selective conditions (McLaughlin, *et al.*, 1991, *Cancer Res.*, **51**, 2242–2245) and cell lines selected by chronic exposure to cisplatin were compared to their respective sensitive parental cell lines. 6/12 independent single-step mutants had elevated p53 levels compared to non-selected A2780 clones. 2/5 of chronically selected cisplatin resistant ovarian lines had elevated p53 levels. Immunoprecipitation of extracts with mutant p53-specific antibodies and direct PCR sequencing of cDNA suggest that this increased p53 is not due to p53 mutations in the resistant lines. Similarly, transfection and expression of a mutant p53 in the sensitive parental line, A2780, does not confer cisplatin resistance or an increase in the frequency of cisplatin resistant mutants occurring in the cell population. However, low doses of cisplatin does induce p53 levels and since this induction has a more prolonged time-course in resistant cells, we suggest that it is the altered induction or stability of the protein that influences response to cisplatin. Studies are underway to examine if inducing p53 can confer resistance and if this affects cell-cycle progression in the different cell lines.

### 12 Immunohistochemical detection of p53 protein as a prognostic marker in mammary carcinoma

D.M. Barnes[1], E.A. Dublin[2], C.J. Fisher[1], D.A. Levison[2] & R.R. Millis[1]

*[1]ICRF Clinical Oncology Unit and [2]Department of Histopathology, UMDS, Guy's Hospital, London, UK.*

An immunohistochemical study for p53 has been carried out on 195 primary breast cancer patients, with a 10-year median follow-up. Anti-p53 polyclonal antibody CM-1 has been used with a peroxidase conjugated streptavidin biotin technique on formalin fixed paraffin embedded sections. The presence of p53 protein was indicated by positive staining of the tumour nuclei. In this pilot study it was found that patients with carcinomas which expressed p53 protein in the majority of their tumour cells (19% of cases) had a considerably worse prognosis than those who did not. The effect of the presence of the protein is seen on disease free interval, overall survival and survival after relapse and is seen in node negative, as well as node positive, patients. Furthermore, the effect is most apparent in patients with infiltrating lobular and grade II infiltrating ductal carcinomas who have a rather heterogeneous clinical behaviour and are difficult to subdivide on the basis of currently available markers. Cox multivariate analysis shows that p53 majority staining is second only to node status in significance of effect on overall survival.

### 13 pRB phosphorylation events in $G_{1A}$ are inhibited during cell cycle arrest by α-IFN

N.S.B. Thomas & A. Bybee

*Department of Haematology, UCMSM, 98 Chenies Mews, London WC1E 6HX, UK.*

The retinoblastoma protein, pRB, is multiply phosphorylated in a cell cycle dependent manner and this is thought to control cell cycle progression. Actively cycling human cells contain partially phosphorylated pRB in early $G_1$ ($G_{1A}$). These intermediate forms of pRB become further phosphorylated as cells pass into late $G_1$ ($G_{1B}$). In contrast, cell cycle arrest of a human B-cell line (Daudi) by α-IFN results in an accumulation of cells in $G_1$ and these cells contain the hypo-phosphorylated form of pRB in both $G_{1A}$ and $G_{1B}$. Hypo-phosphorylated pRB is detectable in $G_1$ within 2–4 h of α-IFN addition, but not in other cell cycle phases even though other events stimulated by α-IFN, such as a decrease in *c-myc* protein levels or induction of the DAI kinase, are not restricted to $G_1$. Since hypo-phosphorylated pRB is thought to prevent cell proliferation, cell cycle arrest induced by α-IFN may be mediated by preventing the initial phosphorylation of pRB during or prior to $G_{1A}$. α-IFN does not increase phosphate turnover on pRB, therefore the decrease in pRB phosphorylation is probably due to decreased kinase activity. One candidate pRB kinase is $p34^{cdc2}/p58^{cyclinA}$. The phosphorylation of $p34^{cdc2}$ and its kinase activity against a pRB peptide is inhibited by α-IFN. Further, α-IFN inhibits $p58^{cyclinA}$ synthesis. In cycling cells $p58^{cyclinA}$ synthesis occurs during $G_{1B}$/early S phase, coincident with the phosphorylation of $p34^{cdc2}$. Thus, $p34^{cdc2}/p58^{cyclinA}$ probably does not cause the initial, partial phosphorylation of pRB in $G_{1A}$, so at least one other unidentified α-IFN-sensitive kinase is needed to 'prime' the phosphorylation of pRB in $G_{1A}$.

Supported by the CRC and Kay Kendall Leukaemia Fund.

### 14 Loss of tumour suppressor genes in prostatic cancer

S.M. Phillips, D. Morton[1], S.J. Lee[2], D.M. Wallace & J.P. Neoptolemos[1]

*Departments of Urology and [2]Pathology, Queen Elizabeth Hospital, Birmingham B15 2TH; and [1]Academic Surgery Unit, Dudley Road Hospital, Birmingham B18 7QH, UK.*

In the UK prostatic cancer accounts for 3% of all deaths in men. Inactivation of tumour suppressor genes is thought to be important in human cancer but very little is known about prostatic cancer. Paired tumour and leucocytes were obtained from 21 men undergoing transurethral resection or obstructive prostate cancer (T2, MO = 4; T2, M1 = 2; T3, MO = 5; T3, M1 = 5; T4, MO = 1; M1 = 4). Loss of heterozygosity was determined by Southern blotting using polymorphic probes in regions of known or suspected tumour suppressor genes.

No deletions were found in chromosome 16p in 16 informative cases. Probing of the RB gene locus revealed three deletions in eight informative cases and three out of seven informative cases using probe EF544 which is close to the APC and MCC loci (π227 revealed no deleteions). Using three probes for chromosome 10p-q (CTB07, EFD75 and D10590) four, three and five deletions were found in 11, 14 and 15 informative cases respectively.

In conclusion these results implicate the RB and APC genes along with a putative suppressor gene (or genes) on chromosome 10 in prostatic carcinogenesis.

### 15 Role of transferrin receptor in estrogen modulated growth regulation in primary breast cancer

V. Makinde[1], S. Dilly[1], C. Corbishley, S. Lowndes, J. Quilliam, R. Sutcliffe, H. Ford & J.-C. Gazet

*Breast Research Unit and [1]Histopathology Department, St George's Hospital, London SW17 0QT, UK.*

The receptor for the iron transport protein transferrin, is commonly expressed on the cells of human carcinoma including breast cancer and breast cancer cell lines. As iron is an essential element of oxidative respiratory enzymes, cells in an actively dividing state require it's incorporation. Growth regulation of breast cancer cells is influenced by estrogen and to a lesser extent, by progesterone. Therefore the present study has examined primary breast tumours for the expression of transferring receptors (TR), estrogen receptor (ER), progesterone receptor (PgR) and epidermal growth factor receptor (EGFR), to see whether TR expression correlated with markers suggesting good growth control (ER, PgR) or with a marker (EGFR) associated with poorly differentiated tumours. Immunocytochemical staining was performed on 114 patients 72 of which were postmenopausal and 42 were premenopausal. Our results show a strong correlation between the expression of TR and ER ($p<0.004$) and between TR and PgR ($p<0.002$), irrespective of the menopause status. There were no significant correlations between EGFR with either TR or ER. The results suggest a possible relationship between TR expression and estrogen mediated growth regulation. For this reason TR may well be a favourable prognostic marker for predicting response to endocrine therapy in addition to its role as a marker for cell proliferation.

### 16 The expression of transforming growth factor-B isoforms in human breast cancers

J. MacCallum[1], J.C. Keen[2], J.M.S. Bartlett[2], A.M. Thompson[1], W.R. Miller[2] & J.M. Dixon[1]

*[1]Department of Surgery, Royal Infirmary and [2]ICRF Unit of Medical Oncology, Western General Hospital, Edinburgh, UK.*

Transforming growth factor (TGF)-βs are a group of regulatory proteins which have in common the ability to transform fibroblasts. In addition, they may have effects on epithelial cell proliferation and it has been proposed that some effects of tamoxifen on human breast cancers may be mediated by induction of the expression of the TGFβs. Three closely-related members of the TGFβ family, $TGF\beta_1$, $TGF\beta_2$ and $TGF\beta_3$ have been identified and the biological activity of these three isoforms may differ. The aim of the present study was to assess whether messenger RNA for the three isoforms of TGFβ could be identified in a series of human breast cancers.

Twenty-four human breast cancers have been studied using RNAase protection assay and specific riboprobes for $TGF\beta_1$, $TGF\beta_2$ and $TGF\beta_3$. Tumours were considered positive for specific RNA expression if bands were identified on autoradiography with molecular weights corresponding to the appropriate full-length transcript. Of the 24 breast cancers, 22 (92%) expressed $TGF\beta_1$, 16 (67%) $TGF\beta_2$ and 21 (88%) expressed $TGF\beta_3$. Sixteen cancers expressed all three isoforms, six expressed one or two of the isoforms (the patterns were different in each of the six) and two cancers did not express any of the TGFβ isoforms although in these two cancers the control γ actin riboprobe was positive. This study is the first to report on the presence of the TGFβ isoforms in human breast cancer. It provides a basis to assess the clinical significance of patterns of TGFβ isoform expression and will allow any changes induced by tamoxifen treatment to be investigated.

### 17 Expression of MRNA for bFGF in human breast cancers and breast cancer cell lines

J.H.R. Winstanley, S. Anandappa, D. Fernig, S.J. Leinster, P.S. Rudland & B.R. Barraclough

*Departments of Surgery and Biochemistry, University of Liverpool, Liverpool, UK.*

It has been suggested that basic fibroblast growth factor (bFGF) is expressed during the growth and development of the normal mammary gland. In addition since bFGF is also a potent angiogenic factor it may play a role in the development of invasive breast cancers. We have therefore investigated the expression of messenger RNA (mRNA) for bFGF in 17 benign breast lesions, 111 invasive carcinomas and in four human malignant breast epithelial cell lines.

Messenger RNA was extracted from snap-frozen biopsy specimens and cell lines by standard methods. Relative levels of bFGF mRNA expression were determined using Northern hybridisation and scanning densitometry. Sixteen (94%) of the benign and 78 (70%) of the malignant lesions had detectable levels of bFGF mRNA (Chi-square $p = <0.02$). The mean and median levels of expression relative to a constitutively expressed marker mRNA were 2 and 5 fold greater in benign than malignant lesions respectively (Mann-Whitney U test $p = 0.001$). Preliminary analysis indicates that bFGF mRNA expression is not related to either tumour size or nodal status. In the malignant cell lines bFGF mRNA and bFGF itself was expressed in two out of four lines examined.

Although there is a significant decrease in the average level of bFGF mRNA expression in malignant compared with benign breast lesions, its expression is maintained in a subgroup of patients similar to its expression in a subgroup of malignant cell lines. Our results therefore suggest the possibility that bFGF may contribute to the development of invasive carcinomas but only in a restricted group of patients.

### 18 Carrageenans: novel, selective growth factor antagonists with anti-proliferative activity

R. Hoffman[1] & D. Paper[2]

*[1]Clinical Oncology and Radiotherapeutics Unit, MRC Centre, Hills Road, Cambridge CB2 2QH, UK; [2]Institute for Pharmacy, University of Regensburg, Universitatstrasse 31, 8400 Regensburg, Germany.*

We have exploited the observation that the polysulphated carbohydrate heparin binds to some growth factors to identify new growth factor antagonists. The activities of compounds were assessed by examining their abilities to reduce the binding of radiolabelled growth factors to cells. From an initial screening programme, carrageenans – polysulphated carbohydrates isolated from red algae – were identified as potent growth factor antagonists. All of the carrageenans tested inhibited binding of basic fibroblast growth factor (bFGF), platelet-derived growth factor (PDGF) and transforming growth factor $\beta 1$ (TGF$\beta 1$) (which all bind to heparin), but the carrageenans did not inhibit binding of insulin-like growth factor 1 (IGF1) or transforming growth factor $\alpha$( TGF$\alpha$) (which do not bind to heparin). The most potent carrageenans for inhibition of binding of bFGF, PDGF and TGF$\beta 1$ were $\tau$-carrageenan, $\kappa$-carrageenan and $\lambda$-carrageenan respectively (IC50 values = 0.3, 0.5 and 15 $\mu$g/ml respectively). I-carrageenan inhibited the proliferation of the FGF-dependent endothelial cell line FBHE as determined in an MTT assay (IC50 approx. 3 $\mu$g/ml) and also inhibited the proliferation of some tumour cell lines. I-carrageenan is of potential use as an anti-cancer agent by inhibiting both autocrine stimulation of tumour cells and by inhibiting the proliferation of capillary endothelial cells – a process required for the neo-vascularisation of tumours.

### 19 Increased malignant cell adhesion to vascular endothelium

D.A. Browell, J.A. Kirby, B.K. Shenton & T.W.J. Lennard

*Department of Surgery, The University of Newcastle upon Tyne, NE2 4HH, UK.*

Increasing adhesion of malignant cells to vascular endothelium is likely to promote intravascular tumour spread and metastasis. We have studied the effect of the supernatant from cultured benign (renal epithelial cells) and malignant (A549 and HT29) cell lines on binding of A549 and HT29 to human umbilical vein endothelial cells (HUVEC). HUVEC were cultured to confluence in a standard medium (M199 containing HEPES buffer endothelial growth cell supplement and 20% (v/v) foetal calf serum). The HUVEC were then cultured for 24 hr with standard media containing 5 or 10% (v/v) supernatant from the benign or malignant cell lines, before $5 \times 10^4$ $^{51}$Cr labelled malignant cells were added for 10 minutes. The monolayers were washed, remaining adherent cells were lysed with 1% Triton, the number of malignant cells bound was judged by $\gamma$-spectrometry. Results were subjected to an analysis of variance. The A549 and HT29 cells bound more avidly to the HUVEC pretreated with supernatant from malignant cells than to the HUVEC with no pretreatment or pretreatment with benign cell supernatant ($p<0.001$). Immunofluorescent studies of the malignant cell supernatant stimulated HUVEC revealed an upregulation of ICAM-1, ELAM-1 and VCAM-1 following a similar period of stimulation ($p<0.001$). This suggests that malignant cells may exert a paracrine effect upon endothelial cells in such a way as to promote malignant cell adhesion and promote the development of metastasis.

### 20 Proliferating cells in human colorectal tumours express the CD44 adhesion molecule

A.M. Abbasi[1], K.A. Chester[1], I.C. Talbot[2], G. Boxer[1] & R.H.J. Begent[1]

*[1]CRC Laboratories, Department of Clinical Oncology, Royal Free Hospital School of Medicine, London NW3 2PF and [2]St. Marks Hospital and ICRF Colorectal Unit, London EC1V 2PS, UK.*

CD44 is an integral membrane protein originally defined by monoclonal antibody F10-44-2 as a glycoprotein surface marker of human T lymphocytes [Dalchau *et al.*, 1980, *Eur. J. Immunol.*, **10,** 745]. At least two distinct functions of CD44 are known. In leucocytes, CD44 is involved in adhesion to high endothelial cells and homing to peripheral lymphoid organs. In epithelial cells CD44 mediates anchorage to hyaluronate in basement membranes. The diverse range of functions associated with CD44 have been partly explained by cDNA sequence data showing that CD44 exists in several different forms, obtained from the same gene by variations in splicing.

Some forms of CD44 have been shown to confer metastatic potential in experimental models [Hofmann *et al.*, 1991, *Cancer Res.*, **51,** 5292; Birch *et al.*, 1991, *Cancer Res.*, **51,** 6660] possibly by mimicking lymphocyte behaviour [Arch *et al.*, 1992, *Science*, **257,** 682]. In normal epithelial cells and CD44 transfected 3T3 cells, however, CD44 expression has been linked to proliferation [Alho & Underhill, 1989, *J. Cell Biol.*, **108,** 1557; Flanagan *et al.*, 1989, *Immunology*, **67,** 167]. We asked whether the expression of CD44 in human cancer was also linked to proliferation.

Flash frozen biopsies were obtained from surgical specimens to three adenomatous polyps and 22 colorectal adenocarcinomas (19 primary and 3 metastatic). CD44 was detected by immunocytochemistry using monoclonal antibody F10-44-2, and proliferative areas defined by immunocytochemistry using Ki-67 monoclonal antibody [Gerdes *et al.*, 1982, *Int. J. Cancer*, **31,** 13] on serial sections.

Results showed that in normal colonic mucosa CD44 was expressed on proliferating cells in the lower third of the epithelial crypt. In tumours CD44 expression was consistently associated with proliferative areas irrespective of tumour stage of differentiation. Non-proliferating areas of the carcinomatous epithelium did not express CD44. There was no apparent association with tumour progression and non-malignant tumours were strongly positive.

CD44 was only associated with proliferation of epithelial cells. Stromal cells in the lamina propria and in the tumours often expressed CD44 but did not appear to be proliferating as defined by Ki-67. This is consistent with observations that the CD44 cell adhesion molecule has distinct functions in different tissues.

Our work demonstrates that F10-44-2 defined CD44 is consistently expressed during proliferation by normal colorectal epithelial cells, non-malignant colorectal tumour cells and colorectal carcinoma cells.

### 21 Activity of type IV collagenase in benign and malignant breast disease

D.W. Miles[1], B. Davies[2], L.C. Happerfield[1], L.G. Bobrow[1], R.D. Rubens[1] & F.R. Balkwill[2]

*[1]ICRF COU, Guy's Hospital and [2]ICRF, LIF, London, UK.*

Invasive carcinoma of the breast is associated with variable loss of basement membrane, of which a major component is type IV collagen. We have localised mRNA and protein expression of the 72 kDa and 92 kDa type IV collagenases using *in situ* hybridisation and immunohistochemistry. Enzyme expression was predominantly stromal and probably associated with macrophages and fibroblasts. Myoepithelial staining was also noted. Zymography allows the quantification of the active and inactive forms of the different molecular weight type IV collagenases. We have examined 11 samples of normal/benign breast tissue and 33 cases of primary breast cancer. Although there was no significant difference in the total amount of 72 + 62 kDa activity in primary breast cancer compared with benign disease (27.6 U v 30.1 U), there was significantly less of the inactive (72 kDa) form in primary tumours 14.04 U v 22.88 U ($p = 0.02$). Conversely the mean amount of the active 62 kDa form was higher in malignant compared to benign disease, though this did not reach standard levels of statistical significance (13.6 U v 7.2 U, $p = 0.06$). The ratio of active to total

72 kDa type IV collagenase was higher in primary breast cancer (0.45 v 0.20, p = 0.003) and this ratio correlated with tumour grade (test for trend $p < 0.0001$). The 92/81 kDa form of the enyzme was found predominantly in grade III tumours with levels in this group being significantly higher than in grade II or grade I tumours (32.3 U v 6.8 U v 1.3 U, p = 0.03 & 0.02, respectively). Our data suggest that in primary breast cancer a greater proportion of the 72/62 kDa enzyme is in the active form and that 92/81 kDa activity is associated with high grade tumours.

### 22 Serum metalloproteinases in prostate cancer

H. Wasan[2], T. Baker[1], S. Tickle[1], A. Docherty[1], J. Flanders[2] & J. Waxman[2]

*[1]Celltech Limited and [2]Oncology, Hammersmith Hospital, London, UK.*

The maxtix metalloproteinases (MMP) have roles in the maintenance of the cellular supporting stroma and in tissue invasion, angiogenesis and metastasis. Novel immunoassays for the detection of interstitial collagenase, stromelysin and tissue inhibitor of metalloproteinase 1 (TIMP 1) have been developed. Levels in 36 patients with prostatic cancer were compared with 57 age matched normal controls and 25 patients with rheumatoid arthritis. There were highly significant differences in serum interstitial collagenase and TIMP-1 levels between cancer patients and normal controls ($P < 0.0001$) and between 19 patients with metastatic disease compared to 19 without ($P < 0.022$). Levels at one year were significantly different in those treated cancer patients in remission versus those progressing ($P < 0.0399$). Serum stromelysin levels were higher in rheumatoid arthritis as compared to cancer patients ($P < 0.0001$), and to normal controls ($P < 0.0001$). Pre-therapy serum TIMP-1 levels predicated patient response at 12 months, as effectively as serum prostatic acid phosphatase. This study provides a basis for the introduction of metalloproteinase inhibitors in the treatment of malignancy.

### 23 Over-expression of metallothionein in malignant melanoma

V.K. Shukla[1], P. Laidler[2], K. Horgan[1], L.E. Hughes[1], M.E. Elms[2], K.W. Schmid[3] & B. Jasani[2]

*Departments of [1]Surgery, [2]Pathology, University of Wales College of Medicine, Cardiff, U.K. and [3]University of Munster, Munster, Germany.*

Metallothioneins (MTs) the ubiquitously expressed low molecular weight proteins with high affinity for heavy metal ions (e.g. zinc, copper, cadmium). MT over-expression in cancer cell lines has been associated with resistance to anticancer drugs and irradiation therapy. Attempts have been made to relate immunohistochemically detectable MT to tumour grade and its tendency to recur. Using a monoclonal anti-MT antibody, E9, we investigated in a retrospective study the MT profile of melanoma.

Paraffin-embedded, formalin fixed tissues were studied from 48 patients with melanoma using standard two step indirect immuno-peroxidase method. The cases comprised 43 superficial spreading melanoma (SSM) and five nodular melanoma (NM) in which the thickness of the tumours was 0.0 to 1.5 mm. The presence of MT was observed in 15 of 43 SSM and all 5 NM ($P < 0.002$). Exposed regions (calf, arm) tended to show MT expression (15/28) more often than other regions (trunk 5/20) ($P < 0.002$). Melanomas less than 0.8 mm in thickness (i.e. with very low potential for metastasis) expressed MT in 29% as compared to 65% in melanomas thicker than 0.8 mm ($P < 0.002$). This study suggests that MT expression is associated with potential for disease progression and thus might be used as a prognostic indicator.

### 25 Regulation of polyamine acetylation by doxorubicin in human breast cancer cells

D.M. Quick & H.M. Wallace

*Departments of Medicine and Therapeutics and Biomedical Sciences, University of Aberdeen, Aberdeen AB9 2ZD, UK.*

The naturally occurring polyamines are essential for cell growth and differentiation. They are found in high concentrations in rapidly growing and cancer cells and intracellular concentrations correlate with cell growth rates, as growth is decreased so polyamine content falls and *vice versa*. Indeed recent evidence suggests that loss of polyamines precedes any inhibition of growth implying a causal role for polyamines in preventing growth. The polyamine content of the cell is decreased by the interconversion of the higher polyamines to either monoacetyl derivatives or smaller amines carrying less charge, a process which may facilitate their excretion. In this study we have investigated the link between growth inhibition and polyamine acetylation, the first step in the interconversion pathway, in a human breast carcinoma cell line using the commonly used anticancer drug, doxorubicin (DOX), to limit cell growth.

T47-D cells were grown under standard cell culture conditions in Dulbecco's medium supplemented with 10% (v/v) foetal calf serum and insulin (8 µg/ml). Polyamine interconversion was measured as spermidine/spermine N-acetyltransferase (SAT) activity. Cell growth was measured as the change in protein content and polyamines were quantified by hplc.

DOX inhibited the growth of T47-D cells with an $ID_{50}$ of about 1 µM. Inhibition of growth was accompanied by a decrease of intracellular polyamine content. Cytosolic SAT activity increased after DOX treatment with the increase being almost exclusively an induction of the $N^1$-SAT, the enzyme responsible for the acetylation of spermine and spermidine in the $N^1$ position. Previous reports have shown similar increases in $N^1$-SAT activity in response to treatment with polyamine analogues however unlike these inducers DOX did not prolong significantly the half-life of the enzyme. DOX itself had no effect on SAT activity when added directly to the *in vitro* assay.

The depletion of cellular polyamines by DOX is due to enhanced acetylation resulting in further degradation and/or excretion. It may be that stimulation of polyamine catabolism, and in particular $N^1$-SAT activity, is an early event in the cellular response to growth limitation by this and other cytotoxic drugs.

### 26 Inhibition of the clonogenic growth of breast cancer cells by tamoxifen and the novel steroidal antioestrogen ICI 182780

D.J. De Friend[1], J. Bell[2], D. Wilkes[3], E. Anderson[2], C. West[3] & A. Howell[1]

*Departments of [1]Medical Oncology, [2]Tumour Biochemistry and [3]Radiobiology, Christie Hospital and Paterson Institute, Manchester M20 9BX, UK.*

Antioestrogen therapy using tamoxifen has an established role in the management of breast cancer. Although used as an oestrogen antagonist, tamoxifen also exhibits intrinsic partial agonist activity, which might potentially reduce its clinical efficacy. ICI 182780 is a novel, steroidal antioestrogen, which is devoid of agonist activity and is currently undergoing clinical trial. We have investigated the antioestrogenic effects of 4-hydroxytamoxifen (Tam) and ICI 182780 (182) on MCF 7 breast cancer cells, using the Courtenay-Mills clonogenic assay, which measures the ability of cancer cells to grow as anchorage independant colonies in soft agar. The standard assay was modified by the use of serum-free media in order to optimise its sensitivity with respect to the effects of endocrine agents.

Using this assay we were able to demonstrate a significant increase in colony forming efficiency (CFE) in the presence of a physiological concentration (1 nM) of Oestradiol ($E_2$) compared to control conditions. This oestrogenic stimulation was inhibited by both ICI 182780 and 4-hydroxytamoxifen in a dose dependant manner at concentrations between 10 nM and 1 μM. However, at the typical pharmacological concentration of 1 μM, ICI 182780 caused a significantly greater inhibition of colony formation than 4-hydroxytamoxifen ($p < 0.001$, Student's t test).

| | *Control* | *$E_2$ (1 nM)* | *$E_2$ + Tam (1 nM) (1 μM)* | $E_2$ + *182 (1 nM) (1 μM)* |
|---|---|---|---|---|
| CFE (%) | 0.374 | 0.648 | 0.07 | 0.007 |

We conclude that, *in vitro*, ICI 182780 produces a more complete inhibition of the growth of oestrogen responsive cells than 4-hydroxytamoxifen and may therefore offer potential therapeutic advantages *in vivo* for the treatment of advanced breast cancer.

### 27 Roles of tumour necrosis factor and serotonin in the antitumour action of the investigational anticancer agent 5,6-dimethyl XAA

B.C. Baguley[1], G.L. Cole[1], L.-M. Ching[1], W.P. Joseph[1], L.L. Thomsen[1], W.R. Wilson[2], L. Zhuang[1] & L.J. Zwi[2]

[1]*Cancer Research Laboratory and* [2]*Section of Oncology, University of Auckland Medical School, Auckland, New Zealand.*

5,6-Dimethyl xanthenone-4-acetic acid (5,6-dimethyl XAA) is a potent analogue of flavone acetic acid (FAA) and has been accepted for potential clinical evaluation by the Cancer Research Campaign, U.K. Like FAA, 5,6-dimethyl XAA appears to exert its antitumour effects indirectly through the stimulation of host responses. 5,6-dimethyl XAA, like FAA, stimulated the release of tumour necrosis factor (TNF), as detected using L-929 cells, and reduced blood flow in s.c. colon 38 and in i.m. mammary MCa4 tumours. 5,6-dimethyl XAA also stimulates nitric oxide formation, induces natural killer activity and increases bone marrow colony forming units in the mouse. These effects presumably all result from secretion of cytokines. The question of whether 5,6-dimethyl XAA stimulates formation of TNF in humans was approached *in vitro* by comparing cultures of mouse (J774) and human (HL-60) macrophage lines. In both of these lines, 5,6-dimethyl XAA stimulated the formation of larger amounts of TNF mRNA than did FAA, and also stimulated at a lower concentration. The question of whether 5,6-dimethyl XAA induces tumour haemorrhagic necrosis through the action of TNF was investigated by taking advantage of the observation that the serotonin antagonist cyproheptadine inhibits the induction of tumour haemorrhagic necrosis by TNF. Co-administration of cyproheptadine significantly inhibited both the induction of tumour haemorrhagic necrosis and the production of nitric oxide in response to 5,6-dimethyl XAA or FAA. Reduction of plasma serotonin concentrations, by pretreatment with reserpine and p-chlorophenylalanine, had a similar but less pronounced effect. It is concluded that both serotonin and TNF are important for the action of 5,6-dimethyl XAA and related compounds.